# Hyponatremia after pediatric surgery: Randomized trial of fluid composition on antidiuretic hormone response

**DOI:** 10.1038/s41390-025-04124-8

**Published:** 2025-05-21

**Authors:** Kazuki Yokota, Hiroo Uchida, Katsunori Manaka, Masaomi Nangaku, Yachiyo Kuwatsuka, Masahiko Ando, Kimitoshi Nishiwaki, Takahiro Hirai, Takahisa Tainaka, Chiyoe Shirota, Wataru Sumida, Satoshi Makita, Hizuru Amano, Akinari Hinoki

**Affiliations:** 1 Department of Pediatric Surgery, Nagoya University Graduate School of Medicine, Nagoya, Japan; 2 Division of Nephrology and Endocrinology, The University of Tokyo, Tokyo, Japan; 3 Center for Advanced Medicine and Clinical Research, Nagoya University Graduate School of Medicine, Nagoya, Japan; 4 Department of Anesthesiology, Nagoya University Graduate School of Medicine, Nagoya, Japan; 5 Department of Rare/Intractable Cancer Analysis Research, Nagoya University Graduate School of Medicine, Nagoya, Japan

## Abstract

**Background:**

We examined the underlying mechanisms of whether hyponatremia could be induced by hypotonic solution administration after the postoperative invasive phase (POIP).

**Methods:**

We included patients who had undergone surgery with expected oral feeding resumption after postoperative day (POD) 3. In this open-label, randomized controlled trial, 100 patients were assigned to three groups by sodium concentrations ([Na]) used for maintenance infusions: 136 mEq/L (ISO) (*n* = 34), 68 mEq/L (HYPO) (*n* = 33), and 34 mEq/L (exHYPO) (*n* = 33). Potassium (20 mEq/L) and glucose (60 g/L) were added to each infusion. Ringer’s solution was used in all groups for the first 12 h postoperatively, then switched to a maintenance solution. Blood samples were drawn and evaluated on POD 3.

**Results:**

Hyponatremia frequencies on POD 3 were 3.6, 18, and 39% in the ISO, HYPO, and exHYPO groups, respectively, with a significant difference between the ISO and exHYPO groups. Additionally, 90% of the patients still had excessive antidiuretic hormone (ADH) secretion on POD 3. There were no remarkable adverse events.

**Conclusion:**

The persistence of surgical stress-induced ADH secretion until POD 3 suggested that hyponatremia was induced by exHYPO administration. However, using Ringer’s solution during the POIP might prevent hyponatremia in HYPO patients.

**Registration number:**

UMIN000029057 (https://www.umin.ac.jp/ctr/index.htm).

**Date of first registration:**

01/11/2017

**Impact:**

- After the postoperative invasive phase, non-osmotic antidiuretic hormone (ADH) secretion due to surgical stress persisted, resulting in an excess ADH state for at least 3 postoperative days (PODs).
- Administration of extremely hypotonic electrolyte infusions under such circumstances might induce hyponatremia.
- Adequate extracellular fluid administration during the invasive phase after major pediatric laparoscopic surgery did not cause further increases in ADH secretion or hyponatremia, even for HYPO maintenance fluid.

## Introduction

Hypotonic electrolyte solutions as pediatric maintenance infusions were first reported by Holliday and Segar^1^ in 1957 and have been used worldwide since then. A solution of approximately 0.2% saline, with the composition based on the calorie and electrolyte content of human and cow milk, is a fairly hypotonic electrolyte solution.^1^ In 2003, Moritz and Ayus described the risks of hypotonic electrolyte solutions as maintenance infusions,^2^ which reportedly caused iatrogenic hyponatremia, a potentially life-threatening condition.

Accordingly, isotonic solutions, such as 0.9% saline, were recommended for maintenance infusions.^2^ In a systematic review of randomized controlled trials (RCTs) that investigated the efficacy and safety of isotonic and hypotonic solutions as maintenance intravenous fluids, Hasim et al.^3^ concluded that isotonic maintenance fluids could prevent hyponatremia, whereas hypotonic infusion increased hyponatremia risk. However, in these RCTs of surgical patients, isotonic or hypotonic infusions were administered as maintenance fluids immediately after surgery,^4–14^ when body fluids are transferred to the third space, causing intravascular dehydration. Therefore, isotonic infusions should be administered during this period, known as the “postoperative invasive phase.” After approximately 12 h of postoperative diuresis, the patient may be switched to receiving maintenance infusion. However, whether a hypotonic or isotonic solution is a better maintenance infusion after the postoperative invasive phase remains unknown.

Inappropriate secretion of antidiuretic hormone (ADH), a condition termed SIADH, has been implicated as a cause of hyponatremia.^2,6–10,15–19^ ADH is secreted by the posterior pituitary gland that regulates fluid volume balance by storing water in the body. Therefore, ADH secretion is regulated by osmotic pressure.^19–21^ However, ADH secretion could also be stimulated by non-osmotic factors, including positive pressure ventilation, stress, nausea and vomiting, pain, hypoglycemia, fever, infection, and various medical conditions and surgeries.^7,10,18^ Patients undergoing inpatient treatment often experience SIADH due to inappropriate non-osmotically stimulated ADH secretion, and hyponatremia is induced upon hypotonic solution administration to patients in this state. Elevated serum ADH in hospitalized patients, both medically and surgically, has been previously confirmed.^7–9,17^

In a 2007 notice, the United Kingdom National Patient Safety Agency stated that 0.18% saline-based infusion products should not be stored in hospital wards.^15^ Additionally, the American Academy of Pediatrics recommended isotonic solutions for pediatric maintenance infusions, according to the 2018 guidelines.^3,22^ However, in Japan and other countries, surgeons still use hypotonic maintenance fluids. Of note, most RCTs to date had an observation period of 12–48 h.^3^

This study aimed to examine and identify the optimal maintenance infusion type after the postoperative invasive phase of major pediatric abdominal surgery. The primary outcome of this RCT was the relationship between maintenance infusion composition and hyponatremia incidence. Our hypothesis was that using hypotonic electrolyte fluids as maintenance fluids post-surgery would increase the incidence of hyponatremia. For the secondary outcome, we examined changes in sodium concentration ([Na]), the relationship between hyponatremia and SIADH, and stimuli of inappropriate ADH secretion.

## Methods

### Study design and ethical consideration

This single-center, open-label, prospective RCT was conducted between January 2018 and March 2021 and was approved by Nagoya University Hospital’s Ethics Committee (No: 2017-0382). This study is registered with the UMIN Clinical Trials Registry (https://www.umin.ac.jp/ctr/index.htm; registration number: UMIN000029057; date of first registration: 01/11/2017). The work described was carried out in accordance with the Declaration of Helsinki. Informed consent was obtained from the patients and/or their legal guardians. Informed assent was given in person to patients aged ≥5 years. Infusions with three different [Na] were prepared as postoperative maintenance solutions for three assigned patient groups. Assigned infusions were administered per the manufacturer’s protocol. Blood and urine samples were collected and evaluated on postoperative day (POD) 3.

### Patient selection and enrollment

Eligible patients were those aged <18 years who had undergone surgical treatment in our department with expected oral feeding resumption after POD 3. This condition was selected because complete control of water delivery by removing the oral intake factor was considered important; this study aimed to determine the effect of different infusion compositions on electrolytes. Since our department follows a protocol for resuming oral intake three days after intestinal anastomosis, target patients were those scheduled to undergo intestinal anastomosis. Patients managed in the neonatal intensive care unit (NICU), those with cardiac, renal, adrenal, endocrine, or severe neurological disease, abnormal electrolytes, abnormal blood pressure, diuretic use, or edema were excluded.

### Randomization

Patients were assigned to three groups (1:1:1) by simple randomization using a computerized block randomization program. The designated the ISO, HYPO, and exHYPO groups received infusions with [Na] of 136, 68, and 34 mEq/L, respectively, for postoperative maintenance solutions. Assignments were made by an independent statistician.

### Procedures

Intervention began after surgery completion. An isotonic Ringer’s acetate solution was used as maintenance infusion for the first 12 h. The composition of Ringer’s solution was as follows: sodium 130 mEq/mL, potassium 4 mEq/mL, calcium 3 mEq/mL, chloride 109 mEq/mL, and acetate 28 mEq/mL. Extracellular fluid administration is desirable because filling intravascular space in the postoperative invasive phase is necessary. The dose was 1.5 times the dose recommended by Holliday and Segar (100, 50, and 20 mL/kg/day for the first 10 kg, the next 10 kg, and the rest of the body weight, respectively).^1^ Twelve hours postoperatively, the vessels were filled, followed by diuresis. At this point, infusions were switched to the assigned maintenance solution. Electrolyte compositions of the maintenance solutions for each group are listed in Table 1. Potassium (20 mEq/L) and glucose (60 g/L) concentrations were similar for all groups. No other electrolytes or trace elements were added. The dose, similar to the one recommended by Holliday and Segar,^1^ was maintained until POD 3. If the stomach was depressurized with an NG tube, the drainage volume was corrected for half saline. If a drain was inserted, the volume drained was given back to the patient using half-normal saline. Depending on vital signs and urine output, the infusion volume could be changed at the attending physician’s discretion, and 85–130% of the planned volume was allowed. To measure white blood cell count (WBC), C-reactive protein (CRP), serum electrolytes, urea nitrogen, creatinine, ADH, adrenocorticotropic hormone (ACTH), plasma renin activity, plasma osmolality, urinary electrolytes, and urine osmolality, blood and urine samples were collected preoperatively, and on POD 1 and 3. Patients were excluded if their condition worsened or if oral intake started earlier owing to a procedural change.

**Table 1:** Patient characteristics, composition of study fluids, and urinary electrolytes.

	ISO	HYPO	exHYPO	*p value*
Na concentration (mEq/L)	136	68	34	
K concentration (mEq/L)	20	20	20	
Glucose concentration (g/L)	60	60	60	
Total, *n*	28	33	33	
Male sex, *n* (%)	13 (46)	14 (42)	12 (36)	0.70^a^
Age at surgery (months), median (range)	16.5 (1, 207)	6 (0, 160)	21 (1, 166)	0.30^b^
Operative time (minutes), median (range)	330 (153, 602)	310 (61, 635)	367 (59, 785)	0.34^b^
Endoscopic surgery, *n* (%)	25 (89)	27 (83)	30 (91)	0.50^a^
Intraoperative fluid volume (mL/Kg/H), median (range)	12.7 (5.5, 29.1)	11.9 (1.5, 32.3)	14.1 (3.3, 48.6)	0.72^b^
Pre- and postoperative weight ratio, median (range)	1.00 (0.88, 1.09)	0.99 (0.92, 1.07)	0.96 (0.92, 1.07)	0.0011^b^
Urinary sodium (mmol/L), median (range)	230 (377, 109)	107 (199, 46)	78 (206, 20)	<0.0001^b^
Urinary sodium – potassium ratio, median (range)	6.5 (13, 1.8)	4.1 (10, 1.8)	3.7 (13, 0.83)	<0.0001^b^

*exHYPO* maintenance fluid with sodium concentration ([Na]) of 34 mEq/L; HYPO: maintenance fluid with [Na] of 68 mEq/L, *ISO* maintenance fluid with [Na] of 136 mEq/L.

Pre- and postoperative weight ratio: the value obtained by dividing the body weight on postoperative day 3 by the preoperative body weight.

^a^Fisher’s exact test.

^b^Kruskal–Wallis test.

### Outcomes

The primary outcome was whether hyponatremia, defined as <137 mEq/L (our institution’s reference value), was induced by the maintenance infusion’s composition. The secondary outcome was the change in [Na] during each infusion. If hyponatremia was induced by the infusion composition, clarification of hyponatremia pathogenesis was considered a secondary outcome. In other words, we examined whether SIADH was involved in hyponatremia and whether inappropriate ADH secretion resulted from a non-osmotic stimulus. After blood collection, ADH was promptly separated from plasma, frozen, and measured by radioimmunoassay. First, we examined whether ADH was oversecreted in relation to plasma osmolality. Since the upper limit of ADH is 0.5×(x-279) pg/mL when the value of plasma osmolality is x, this convention was adopted.^23^

### Statistical analysis

#### Sample size determination

In our previous retrospective studies, hyponatremia was identified in 5% of HYPO and 52% of exHYPO patients.^24^ As most previous studies reported the hyponatremia frequency of HYPO patients as 10–20%,^4,5,10,14,18^ we hypothesized that a slightly smaller difference might exist between two groups; 10 and 40% of HYPO and exHYPO patients, respectively, would have hyponatremia with an alpha of 0.05 and 80% power, and the required sample size was 31 cases per group. Therefore, the total number of samples required for the three groups was 93, and our planned number of samples was 100.

#### Primary outcome

The characteristics of the three patient groups were compared using the Kruskal–Wallis and Fisher’s exact tests. Hyponatremia frequencies of the three groups were compared by Fisher’s exact test. Multiple-comparison results from Fisher’s exact test were corrected by Bonferroni method, and pairwise comparisons were made between ISO and HYPO, ISO and exHYPO, and HYPO and exHYPO, for which *p* = 0.0167 was considered statistically significant. Hyponatremia frequencies on POD 3 and 95% confidence intervals (CI) based on binomial distribution were calculated for each group.

#### Secondary outcome

Adjusted mean and 95% CI of the change in [Na] over time from the preoperative status to POD 1 and 3 were calculated at each time point using a linear mixed model with fixed effects for the interaction between treatment group and time point. For between-group comparisons, analyses were performed using linear mixed models, and changes in [Na] were compared after multiplicity correction with the Tukey–Kramer method.

Correlation analyses of ADH values on POD 3 with other indices were performed. First, the numbers of cases of ADH hypersecretion in the ISO, HYPO, and exHYPO groups were calculated and compared using Fisher’s exact test. Absolute ADH values on POD 3 were compared using one-way analysis of variance (ANOVA), and the Bonferroni method was used to compare the three groups. Multivariate linear regression analysis was performed on absolute ADH values on POD 3 to examine inappropriate ADH secretion using each of the following variables as covariates: including allocation group, age (<1 vs. 1–6 vs. 6–10 vs. >10 years), endoscopic surgery (open vs. laparoscopic), surgery duration (minutes), blood loss, WBCs and CRP on POD 1 and 3, ACTH, plasma renin activity, Fractional Excretion of Sodium (FENa), and Fractional Excretion of Urea Nitrogen (FEUN), were used. From these variables, the ones with *p* < 0.1 in univariate regression were used in multivariate analysis. Allocation groups were entered into multivariate models.

## Results

### Patients and controls

**Fig. 1:**
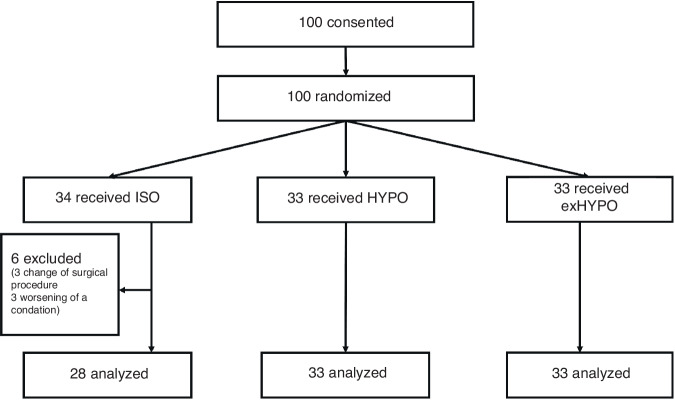
Flow diagram of the selection of the study participants.

Between January 2018 and March 2021, consent was obtained from 100 participants (Fig. 1), who were randomized and assigned to ISO (*n* = 34), HYPO (*n* = 33), and exHYPO (*n* = 33) groups. Six ISO patients were excluded because of a procedural change (*n* = 3), diuretics administration (*n* = 2), or massive intraoperative bleeding requiring multidisciplinary care (*n* = 1). No HYPO or exHYPO patients were excluded. Table 1 shows patient characteristics in each group. For the ISO, HYPO, and exHYPO groups, male ratios were 46, 42, and 36%, respectively (*p* = 0.70), endoscopic surgery rates were 89, 83, and 91%, respectively (*p* = 0.50), median ages at surgery were 16.5, 6, and 21 months, respectively (*p* = 0.30), and median surgery durations were 330, 310, and 367 min, respectively (*p* = 0.34), without any significant differences among the groups. Regarding surgical procedures, 39, 17, 10, 9, 4, and 15 patients underwent radical congenital biliary dilatation, radical biliary atresia, fundoplication, radical surgery for Hirschsprung’s disease, colostomy, and other procedures, respectively. The intraoperative fluid volume and the pre- and postoperative weight ratio were shown in Table 1. “Weight ratio” was defined as the value obtained by dividing the body weight on POD 3 by the preoperative body weight. The median intraoperative fluid volumes in the ISO, HYPO, and exHYPO groups were 12.7, 11.9, and 14.1 mL/Kg/H, respectively (*p* = 0.72), while the weight ratios were 1.00, 0.99, and 0.96 (*p* = 0.0011). Additionally, the planned maintenance fluid volume was modified in five cases. One case in the ISO group was adjusted to 130%, one case in the HYPO group to 130%, and three cases in the exHYPO group, with one adjusted to 120% and two to 130%.

### Primary outcome

Based on blood samples on POD 3 (Table 2), ISO, HYPO, and exHYPO induced hyponatremia in 3.6, 18, and 39% of the patients, respectively, with a significant difference among the groups (*p* = 0.002). Pairwise comparison showed a significant difference only between the ISO and exHYPO groups (*p* = 0.001). However, no patients showed hypernatremia until POD 3.

**Table 2:**
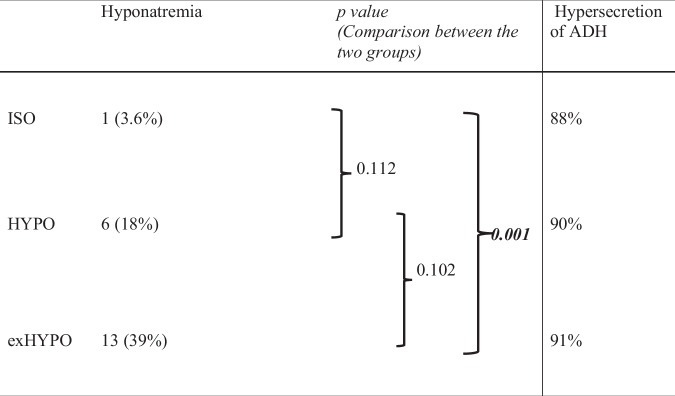
Frequencies of hyponatremia and hypersecretion of ADH at postoperative day 3.

There was a significant difference between the three groups by Fisher’s exact test for hyponatremia frequency (*p* = 0.002) and no significant difference for hypersecretion of ADH (*p* = 0.95).

*ADH* antidiuretic hormone, *exHYPO* maintenance fluid with sodium concentration [Na] of 34 mEq/L, *HYPO* maintenance fluid with [Na] of 68 mEq/L, *ISO* maintenance fluid with [Na] of 136 mEq/L.

### Secondary outcome

**Fig. 2:**
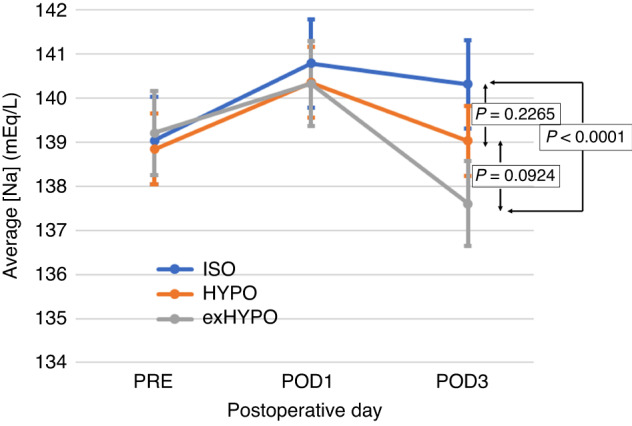
Changes in serum sodium concentrations [Na] in the exHYPO, HYPO, and ISO groups (maintenance fluids with [Na] of 34, 68, and 136 mEq/L, respectively). exHYPO maintenance fluid with sodium concentration [Na] of 34 mEq/L, HYPO maintenance fluid with [Na] of 68 mEq/L, ISO maintenance fluid with [Na] of 136 mEq/L

Figure 2 shows [Na] trends for different maintenance infusion compositions. Three groups had the same conditions preoperatively and up to 12 h postoperatively, including the administered maintenance infusion type, and there were no differences preoperatively or on POD 1. However, average [Na] values in the ISO, HYPO, and exHYPO groups on POD 3 were 140.32, 139.03, and 137.61, respectively, with a significant difference between the ISO and exHYPO groups (*p* < 0.0001). Due to using Ringer’s solution as a maintenance fluid for 12 h postoperatively in three groups, [Na] values on POD 1 were elevated in all groups as compared to preoperative values. However, the exHYPO group did not retain [Na] on POD 3. Table 1 shows urinary sodium excretion and sodium-potassium ratios, which were significantly higher in the ISO group. No difference in sodium-potassium ratios between the HYPO and exHYPO groups were observed.

The cause of hyponatremia was examined. Common conditions that may cause hyponatremia include vomiting, diarrhea, diuretic use, renal disease, heart failure (HF), adrenal insufficiency, hyperglycemia, and SIADH. Patients using diuretics and those with renal disease or HF were excluded. Patients were thoroughly monitored for diarrhea and other fluid loss, and adrenal insufficiency and hyperglycemia were examined using blood samples to ensure no problems existed. Therefore, no patients met the aforementioned abnormal conditions. Regarding urinary findings, no patients had a urinary [Na] <20 mEq/L, and only one had a urine osmolality <100 mOsm/kg. Frequencies of cases with excess ADH secretion over the reference value were 88, 90, and 91% in the ISO, HYPO, and exHYPO groups, respectively, with no significant difference between the groups (*p* = 0.950, Table 2). We next examined factors affected by ADH hypersecretion. In univariate analysis, only age and ACTH had *p* < 0.1. In multivariate analysis with these factors, only ACTH levels showed significant differences. After adding allocation groups to these results, the only factor significantly correlated with ADH secretion was ACTH in exHYPO patients (95% CI: 0.039–0.1303, *p* < 0.001, Table 3).

**Table 3:** Multivariate analysis of correlation with ADH.

	Coefficient	95% CI		*p value*
Age				
≤5 (years)	−3.23	−9.43	2.98	0.3
6–9 (years)	−3.39	−11.1	4.27	0.38
≥10 (years)	−3.38	−12.3	5.49	0.45
ACTH/ISO	−0.0038	−0.038	0.03	0.82
ACTH/HYPO	0.084	−0.0061	0.17	0.067
ACTH/exHYPO	0.085	0.039	0.13	<0.001

*ACTH* adrenocorticotropic hormone, *ADH* antidiuretic hormone, *CI* confidence interval, *exHYPO* maintenance fluid with sodium concentration [Na] of 34 mEq/L, *HYPO* maintenance fluid with [Na] of 68 mEq/L, *ISO* maintenance fluid with [Na] of 136 mEq/L.

## Discussion

In this study, using extremely hypotonic electrolyte solutions as maintenance fluids was a risk factor for hyponatremia, even after adequate extracellular fluid administration during the postoperative invasive phase. Furthermore, 12 h administration of Ringer’s solution during the postoperative invasive phase could maintain [Na] even for HYPO maintenance solution. Inappropriate non-osmotically stimulated ADH secretion caused hyponatremia and persisted until POD 3 after major pediatric abdominal surgery. If exHYPO maintenance infusion continued to be used under such circumstances, [Na] would not be appropriately maintained and would gradually decline.

In all previous RCTs, using hypotonic (half-saline) fluids as maintenance infusions was a hyponatremia risk factor. However, in these reports, hypotonic electrolyte maintenance infusions were administered immediately after surgery.^4–14^ In this study, isotonic infusion was used to compensate for postoperative deficiencies in each group for up to 12 h postoperatively during the invasive phase before switching to the maintenance infusion. Hyponatremia was induced upon administering exHYPO maintenance fluid, even during periods of water balance. A significant difference in hyponatremia frequency was only observed between our ISO and exHYPO groups. However, many previous studies reported that ISO maintenance fluid should be used because it also induces hyponatremia without maintaining [Na],^5,7,9,10,15,25^ which might be attributable to [Na] maintenance in HYPO by extracellular fluid administration up to 12 h postoperatively.

The secondary outcome was hyponatremia, indicating that inappropriate ADH secretion persisted during the postoperative invasive phase. Even on POD 3, 90% of the patients had ADH hypersecretion. ADH secretion reportedly peaked at POD1^7,9,10,17,26^ but continued to be excessive as it gradually decreased thereafter. While many reports previously analyzed absolute ADH values, our study used a reference value calculated from the osmotic pressure to improve the sensitivity for detecting the excess state. Following continuous exHYPO infusion, [Na] increased on POD 1 but continued to decrease until POD 3, when it was significantly different from the ISO group’s value. While it is known that [Na] levels gradually decrease when an exHYPO maintenance infusion is administrated,^5,15^ it was not known that they would decrease again after having once increased due to the administration of Ringer’s solution.

Regarding stimuli of ADH secretion, only ACTH correlated with ADH. Inflammatory markers (WBC and CRP) and intensity of operative invasiveness (operation time and blood loss) did not correlate with ADH secretion, suggesting that ADH secretion was stimulated by inflammation and surgical stress, but not proportional to their intensity. In other words, ADH was oversecreted regardless of the degree of operative invasiveness. Healthy patients with an American Society of Anesthesiologists physical status of I or II were reportedly less likely to have medically induced hyponatremia.^12^ Nonetheless, their ADH secretion was stimulated.

Among existing osmotic and non-osmotic stimulatory mechanisms of ADH secretion, corticotrophin-releasing hormone (CRH) is involved in non-osmotic stimulation. CRH and ADH reportedly co-localized in the same neurons, and both were induced by stress or inflammation.^21,27^ Therefore, elevated ACTH might be associated with non-osmotically stimulated ADH secretion. In this study, ACTH correlated with ADH secretion, indicating that ADH secretion’s stimulus was derived from CRH. In other words, non-osmotic stimulation, such as surgical stress, was confirmed as ADH secretion’s stimulus on POD 3. When the infusion group was included in regression analysis, a correlation was confirmed only for exHYPO, indicating that osmotic stimulation might have occurred along with non-osmotically stimulated ADH secretion due to higher plasma osmolality in ISO and HYPO groups. Alternatively, exHYPO might be involved in non-osmotic stimulation. Fluid type, rather than fluid restriction, was reportedly a risk factor for hyponatremia.^7,9^ Administration of exHYPO might not fill intravascular space, and hypovolemia might non-osmotically stimulate ADH secretion.^10,26,28^ In this study, there was no difference in intraoperative fluid volume between the groups, and no variation in surgical procedures was observed; however, a slight decrease in body weight was noted in the exHYPO group. The reason for this weight loss is unknown, but it may suggest a reduction in blood volume.

This study excluded all common causes of hyponatremia, except for SIADH. Additionally, despite hyponatremia, urinary [Na] was concentrated and confirmed to be >20 mEq/L. Reducing infusion volume reportedly avoided hyponatremia risk even if hypotonic fluids were administered,^29,30^ which are consistent with SIADH pathogenesis. In summary, our results suggested that hyponatremia was induced upon exHYPO maintenance solution administration under SIADH condition because non-osmotic ADH secretion during the postoperative invasive phase, caused by inflammation and surgical stress, remained until POD 3. However, after the postoperative invasive phase, ADH secretion decreased. Therefore, [Na] could be maintained even when HYPO maintenance infusion was administered during this period. The most significant difference between our results and previous findings was extracellular fluid infusion immediately after surgery.

Which treatment should be administered after the invasive phase (ISO or HYPO)? While previously reported risks of ISO administration included hypernatremia, edema exacerbation, weight gain, and excess chloride administration-associated metabolic acidosis, no significant differences in their frequencies were observed in some studies.^3,5,10,11,18,31^ No adverse events, such as hypernatremia, were observed in ours. However, urinary sodium excretion and sodium-potassium ratio were significantly higher in the ISO group, and there are concerns about their burden on the cardiovascular system and kidneys.^32,33^ Therefore, if [Na] could be maintained with HYPO, HYPO might be recommended after the invasive phase with strict electrolyte management. Nevertheless, although there is no statistically significant difference, the incidence of hyponatremia in the HYPO group is high, so caution should be exercised as it may be potentially harmful. As a precaution, because saline or Ringer’s solution alone lacks sufficient potassium,^11^ the required potassium amount must be added to the maintenance solution. In this study, an appropriate potassium amount was added to all groups.

This study had some limitations. First, it was an open-label, single-center study, in which physicians, nurses, pharmacists, and other staff members in charge of patient care were not blinded to the infusion type. The ideal double-blind design was not possible owing to institutional setup. Further, considering SIADH, a study group with varied infusion volumes might have been more desirable. Second, selection bias might have occurred because oral intake was prohibited during the study period and the study had more exclusionary conditions to eliminate various factors. Third, although our study included major pediatric abdominal surgeries, nearly 90% of them were laparoscopic, which might have resulted in a different outcome as compared to previous findings. Fourth, ADH is unstable, and there are limitations to the reliability of the measurements.

In conclusion, after the postoperative invasive phase, non-osmotic ADH secretion due to surgical stress persisted, resulting in an excess ADH state for at least 3 PODs. Administration of extremely hypotonic electrolyte infusions under such circumstances might induce hyponatremia. Adequate extracellular fluid administration during the invasive phase after major pediatric laparoscopic surgery did not cause further increases in ADH secretion or hyponatremia, even for HYPO maintenance fluid.

## Supplementary information

Consort check list.

## Data Availability

The datasets used and/or analyzed during the current study available from the corresponding author on reasonable request.
